# Recent Advances in Design and Preparation of Polymer-Based Thermal Management Material

**DOI:** 10.3390/polym13162797

**Published:** 2021-08-20

**Authors:** Hongli Zhang, Tiezhu Shi, Aijie Ma

**Affiliations:** School of Materials Science and Chemical Engineering, Xi’an Technological University, Xi’an 710021, China; zhanghongli@xatu.edu.cn (H.Z.); s1903310259@163.com (T.S.)

**Keywords:** thermal management material, thermal conductivity, thermally conductive polymer composites, phase-change material

## Abstract

The boosting of consumer electronics and 5G technology cause the continuous increment of the power density of electronic devices and lead to inevitable overheating problems, which reduces the operation efficiency and shortens the service life of electronic devices. Therefore, it is the primary task and a prerequisite to explore innovative material for meeting the requirement of high heat dissipation performance. In comparison with traditional thermal management material (e.g., ceramics and metals), the polymer-based thermal management material exhibit excellent mechanical, electrical insulation, chemical resistance and processing properties, and therefore is considered to be the most promising candidate to solve the heat dissipation problem. In this review, we summarized the recent advances of two typical polymer-based thermal management material including thermal-conduction thermal management material and thermal-storage thermal management material. Furtherly, the structural design, processing strategies and typical applications for two polymer-based thermal management materials were discussed. Finally, we proposed the challenges and prospects of the polymer-based thermal management material. This work presents new perspectives to develop advanced processing approaches and construction high-performance polymer-based thermal management material.

## 1. Introduction

With the innovation of the electronics industry and the development of 5G technology, electronic devices tend to be miniaturized, highly integrated, and multi-functional. The power density of electronic devices therefore need to be increased continuously, producing a large amount of excess heat that cannot be effectively dissipated [1,2]. To improve the working performance and the lifetime of electronic devices, developing thermal management material with efficient heat dissipation capabilities is necessary. Traditional thermal management materials including metal and ceramic material possess high thermal conductivity, but involve poor processing performance, high density, and high cost, which limit their applicability [3]. Polymers have superior mechanical properties, electrical insulation, chemical resistance, light weight, and excellent processability [4], which play an important role in the fields of electronic devices, aerospace, transportation, etc. The polymer-based thermal management material has therefore aroused great interest in researchers [5].

According to the mechanism of heat dissipation, thermal management material could be classified into thermal-conduction thermal management material and thermal-storage thermal management material. On one hand, the intrinsic thermally conductive polymers feature special phonon transfer structure; the thermally conductive polymer composites contain various thermally conductive fillers, they therefore exhibit high thermal conductivity that can be used to quickly conduct excess heat of electronic devices to the surrounding environment, and they are commonly used thermal-conduction thermal management material [6,7]. On the other hand, the latent heat energy-storage technology using the phase-change process of phase-change material (PCM) to store and release heat, possesses high energy-storage density and approximately isothermal thermal energy-storage process, which also has been widely applied in various thermal management applications [8,9]. Due to its high energy-storage density and thermal conductivity, PCM are considered to be a promising candidate for the preparation of thermal-storage thermal management material [10,11]. However, it remains an alluring goal to construct the thermal-conduction thermal management material and thermal-storage thermal management material with higher thermal conductivity and more effective heat dissipation. Therefore, reviewing the physical fundamentals and recent advances in preparation of the two types of thermal management material is mandatory, which presents new perspectives to design high-performance thermal management material and enhance their thermal dissipation ability.

**Figure 1:**
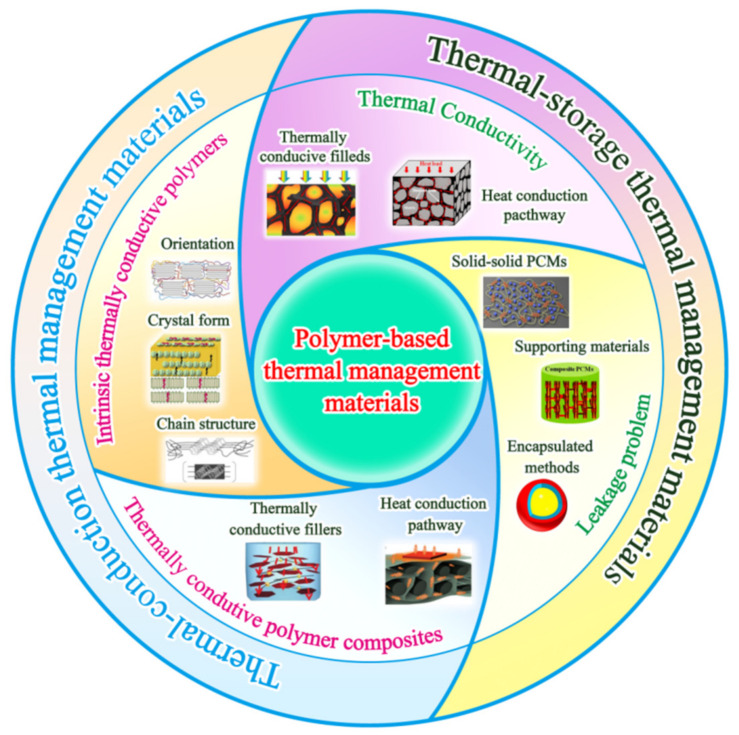
Schemes of two important types of polymer-based thermal management material reported in this review.

To the best of our knowledge, many reviews focusing on thermally conductive material have been reported. However, a conclusive review regarding polymer-based thermal management material is lacking. In this paper, we reviewed the two important types of polymer-based thermal management material reported in recent years (Figure 1). Additionally, we discussed the factors that affect the thermal conduction of polymer-based thermal management material. The feasible structural design and processing strategies to improve their thermal conductivity were presented. Furthermore, the structure design and processing strategy for the preparation of thermal-storage polymer-based thermal management material with high energy-storage density and fast thermal response rate were summarized. We also discussed the typical applications of polymer-based thermal management material. Finally, the challenges and prospects of the polymer-based thermal management material were proposed.

## 2. Intrinsic Thermally Conductive Polymers

**Figure 2:**
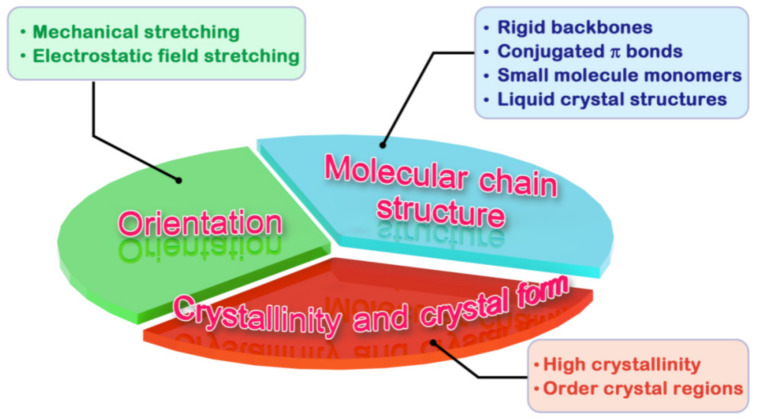
Thermal conductivity of intrinsic thermally conductive polymers depends on various factors that need to be considered.

On the basis of the preparation process, the thermally conductive polymer is usually categorized into the intrinsic thermally conductive polymers and the thermally conductive polymer composites [10]. The thermal conductivity of solid material is mainly determined by the thermal conduction of phonons (energy quanta of lattice vibrations) and free electrons [11]. For polymers, regarded as thermal insulators, the thermal conductivity of polymers is dominated by the contribution of phonons, while in metals, the contribution from electrons is much greater than that of phonons [5,12]. According to Debye’s assumptions, the thermal conductivity (*K*) of polymers could be expressed as Equation (1):

(1) $$K=\frac{C_{P}\nu l}{3}$$

where *C_p_* is the specific heat capacity per unit volume, *ν* is the phonon velocity and *l* represents the phonon mean free path. For bulk polymers, the thermal conductivity is extremely low (<0.5 Wm^−1^K^−1^) [13]. The polymer chains of bulk polymer are in a twisted and random orientation state, and there are a great number of crystal–amorphous interfaces, chain ends, defects and voids, all of which result in strong phonon scattering thus extremely low thermal conductivity. Conversely, by molecular dynamics simulations, Henry and Chen calculated that the thermal conductivity of single polyethylene (PE) chains is up to 350 Wm^−1^K^−1^ [14]. For most polymers, *v* and *C_p_* are almost the same as those of single chain, thus the difference of thermal conductivity between single-chain and bulk polymer is caused by the value of *l* [15]. Therefore, in the process of polymerization and processing, transforming the structure of polymer chains into regular arrangement in a certain direction is the key to obtain intrinsic thermally conductive polymers. Recently, many studies have demonstrated that changing the molecular chain structure, increasing the crystallinity, transforming the crystal form and enhancing orientation of molecular chains are effective strategies to improve the thermal conductivity of the intrinsic polymers [16] (Figure 2).

### 2.1. Molecular Chain Structure

**Figure 3:**
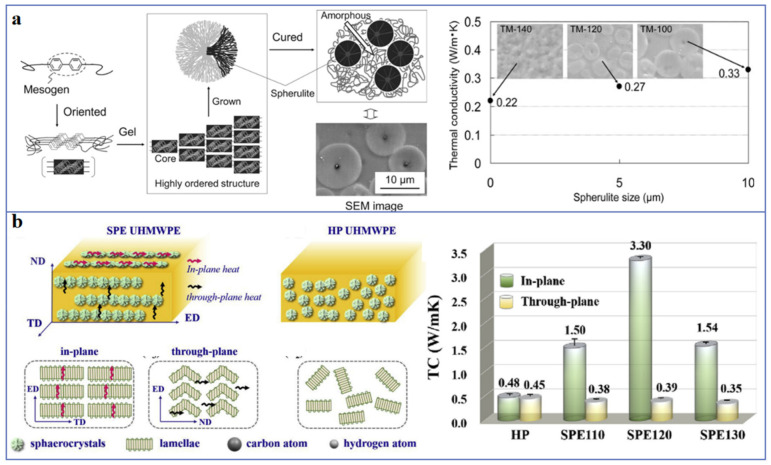
Schematics of the influence of (**a**) molecular chain structure [20] (Copyright (2012) with permission from Elsevier Ltd.) and (**b**) crystal morphology [22] on thermal conductivity (Copyright (2019) with permission from Elsevier Ltd.).

Polymers with rigid backbones have higher thermal conductivity, such as polyphenylene sulfide (PPS) [17], because the rigid backbone can inhibit the rotation of polymer chains and ameliorate the transmission of phonons. Conjugated π-bonded polymers also exhibit higher thermal conductivity, such as polyacetylene, polyaniline, polypyrrole, polythiophene, etc. This is due to the phonon heat conduction mechanism as well as the electron heat conduction mechanism. Zheng et al. [18] synthesized copolymers of 3-alkylthiophene and 3-alkoxythiophene with different p-π conjugation degree by oxidative polymerization. The results of laser measurement and molecular dynamics simulation show that the copolymers with high p-π conjugation degree have thermal conductivity as high as 0.374 Wm^−1^K^−1^. In addition, the introduction of small molecule monomers, liquid crystal structures or other regular structures in the synthesis of polymers can improve the microscopic order of the polymers, thus enhancing thermal conductivity [19]. As shown in Figure 3a, Song et al. [20] reported that the mesogenic epoxy resin maintains a high thermal conductivity of 0.33 Wm^−1^K^−1^, which is 1.7 times higher than that of amorphous epoxy resin, and the mesogenic epoxy resin with spherulite structure have a higher thermal conductivity of 1.16 Wm^−1^K^−1^. Recently, Ruan et al. [21] synthesized liquid crystalline polyimide (LC-PI) films using phthalimide groups as the mesomorphic units. The obtained LC-PI films with microscopically ordered molecular chains exhibit high thermal conductivity of 2.11 Wm^−1^K^−1^ (in-plane) and 0.32 Wm^−1^K^−1^ (through-plane).

### 2.2. Crystallinity and Crystal Morphology

Crystallization can increase the degree of structural order in polymer, so the thermal conductivity of crystalline polymers is generally higher than that of amorphous polymers, and the thermal conductivity of crystalline polymers increases with the increase of crystallinity [23,24]. Bai et al. [25] discussed the effect of crystallinity degree of poly-l-lactide (PLLA) on thermal conductivity. The results show that the thermal conductivity of PLLA increases from 0.16 Wm^−1^K^−1^ (amorphous PLLA) to 0.2 Wm^−1^K^−1^ when the crystallinity is 56%. Because the crystallinity of most polymers is less than 100%, there will inevitably be an interface between crystal and amorphous region, which will lead to phonon scattering and reduce the thermal conductivity. For instance, Huang et al. [22] reported that the ultrahigh molecular weight polyethylene (UHMWPE) has high thermal conductivity of 3.30 Wm^−1^K^−1^, which is due to the reduction of interface between crystals and amorphous regions through the formation of cylindrical crystals and highly oriented lamellae (Figure 3b). In addition, some studies show that the crystal morphology also has an important influence on the thermal conductivity of polymer. For example, the thermal conductivity of high-density polyethylene (HDPE) and UHMWPE with extended-chain crystals is higher than that of folded-chain lamella crystals [24].

### 2.3. Orientation of Molecular Chains

As mentioned above, the main factor causing the low thermal conductivity of the polymer is the random arrangement of the polymer molecular chains, because it is easier to conduct heat along the molecular chains rather than lateral direction. Therefore, the anisotropic thermal conductivity (especially the enhanced thermal conductivity along the stretching direction) can be achieved by aligning polymer molecular chains along a specific direction by mechanical stretching. So far, many experiments have studied the effect of orientation on the thermal conductivity of polymers, including crystalline and amorphous polymers [17,26,27].

For crystalline polymers, many studies have focused on polyethylene (PE). For example, Choy et al. [28] reported that when the drawing ratio is in the range of 1–25, the thermal conductivity of HDPE parallel to the stretching direction is much higher than that of HDPE perpendicular to the stretching direction in the temperature range of 120–320 K. To be specific, when the drawing ratio is 25, the thermal conductivity of HDPE parallel to the drawing direction is up to 8.5 W^−1^mK^−1^ (120 K) and 14 Wm^−1^K^−1^ (320 K). Choy et al. [29] further studied the effect of different drawing ratios (in the range of 1–350) on the thermal conductivity of PE, and the ultra-drawing PE fibers with the drawing ratio of 350 has a high thermal conductivity of 41.8 Wm^−1^K^−1^ in the drawing direction. This can be explained by the fact that when the drawing ratio increases, the crystal lamellae are broken into small crystal blocks and then rearranged to form microfibers; after further stretching, the microfibers are deformed into ordered long extended-chain crystals or even needle-like crystals. Shen et al. [30] also fabricated a series of ultra-stretched PE nanofibers, whose drawing ratio (60–800) is higher than that of Choy [29], and the highest thermal conductivity reaches 104 Wm^−1^K^−1^, which is almost close to the properties of PE single crystals.

## 3. Thermally Conductive Polymer Composites

**Figure 4:**
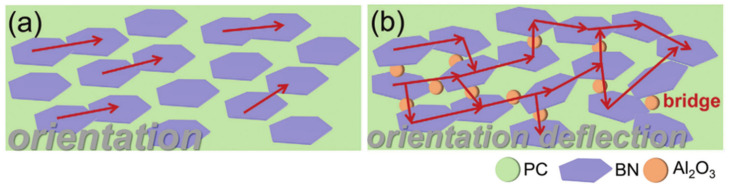
Schematic representation of heat flux in polycarbonate/BN/Al_2_O_3_ composites (**a**) oriented BN platelets for high in-plane thermal conductivity, (**b**) oriented BN platelets with spherical Al_2_O_3_ acting as abridge for both high in-plane and through-plane thermal conductivity [32] (Copyright (2021) with permission from Wiley Periodicals, Inc.).

The preparation process of intrinsic thermal conductive polymer is complex, the cost is high, and large-scale production is difficult to realize. Therefore, it is a simple and economical strategy to add thermally conductive fillers to polymer matrix to prepare thermally conductive polymer composites [14]. Li et al. [31] prepared boron nitride nanosheets (BNNSs)/polydimethylsiloxane (PDMS) composites in a high-speed mixer with a rotating speed of 2500 rpm. The thermal conductivity of the cured composites with a filling amount of 35 wt% is 1.16 Wm^−1^K^−1^, which is about 5 times higher than that of pure PDMS. Bai et al. [32] fabricated polycarbonate/BN/Al_2_O_3_ ternary composites by constructing dense packing structure of hybrid fillers, in which the content of BN and Al_2_O_3_ is determined as 20 wt% and 40 wt% (Figure 4). Consequently, the composite exhibits high in-plane and through-plane thermal conductivity of 1.52 and 1.09 Wm^−1^K^−1^, respectively.

### 3.1. Thermally Conductive Fillers

**Figure 5:**
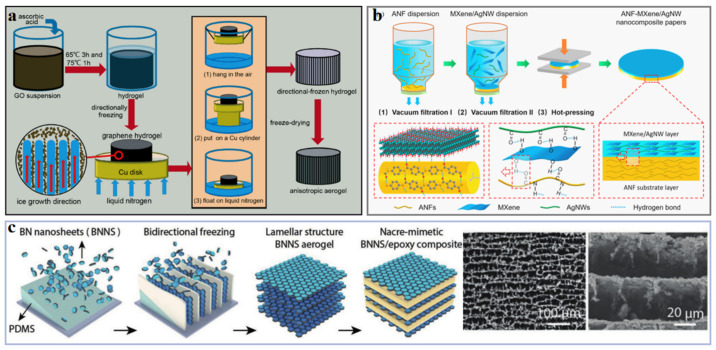
Schematic diagrams of thermally conductive polymer composites with high thermal conductivity by adding (**a**) carbon material [53] (Copyright (2018) with permission from Elsevier Ltd.), (**b**) metals [55] (Copyright (2020) with permission from American Chemical Society) and (**c**) ceramics [67] (Copyright (2019) with permission from Wiley Periodicals, Inc.).

The thermally conductive fillers commonly used in polymers to improve thermal conductivity mainly include carbon material, metals and ceramics (Figure 5 and Table 1). The addition of metal fillers and some carbon material (such as graphite) endows the composite polymers with the integrated thermal conduction of electrons and phonons, which helps to greatly improve the thermal conductivity [33]. Unfortunately, the increase of electrical conductivity and/or the decrease of dielectric breakdown voltage may limit the applications of composite polymers in some cases [34,35]. Ceramic fillers show excellent electrical insulation properties (e.g., hexagonal-BN maintains a high band gap of 5.2 eV and a low dielectric constant of 3–5 [36]), which can be used to prepare electrical insulation and thermally conductive composite polymers.

#### 3.1.1. Carbon Material

Various carbon material, such as carbon nanotubes (CNTs) [38,39], carbon fibers (CFs) [40,41], graphite [42], expanded graphite (EG) [43,44], graphene oxide (GO) [45,46], graphene nanoplatelet (GNPs) [47,48], etc., have been widely prepared and used in polymer composites to improve thermal conductivity. Li et al. [49] prepared EG aerogels in a closed container by liquid expansion method, and then introduced epoxy resin into aerogels by vacuum infiltration process. The resultant composite displayed high through-plane thermal conductivity of 4.14 Wm^−1^K^−1^ when the EG content is 0.91 vol%. Liang et al. [50] built a CNTs-bridged reduced GO network by hydrothermal treatment, and then introduced it into epoxy resin through vacuum-assisted infiltration method to obtain electrical insulation composite with in-plane thermal conductivity of 0.69 Wm^−1^K^−1^. It is pointed out that the carbon-based aerogel with three-dimensional interconnected structure can greatly enhance the thermal conductivity of polymer composites under low load content. In recent years, it has attracted wide interest of researchers [51,52]. For example, Li et al. [53] fabricated a graphitized graphene aerogels (GGA) with highly aligned graphene network by directional-freezing of graphene oxide hydrogel followed freeze-drying and subsequent graphitization at 2800 °C. The epoxy/GGA composites with GGA content of only 1.5 wt% was prepared by vacuum-assisted impregnation method, and the through-plane thermal conductivity of the graphite is 6.57 Wm^−1^K^−1^.

#### 3.1.2. Metals

Metal has been widely studied to improve its thermal conductivity due to its inherent properties. So far, Ag [54,55], Cu [56] and Ni [57] are the three most commonly used metals to enhance the thermal conductivity of composite polymers. Huang et al. [58] prepared ferroelectric polymer nanocomposites composed of poly(vinylidene fluoride) and Ag nanoparticles. When the content of Ag nanoparticles is 20 vol%, the thermal conductivity of the nanocomposites is as high as 6.5 Wm^−1^K^−1^. Bhanushali et al. [59] fabricated copper nanowires (CuNWs) with interconnected network structure by freeze-casting into a monolithic sponge. Under the low CuNWs load of 1.8 vol%, the thermal conductivity of flexible silicone rubber-based composites impregnated copper nanowires is 3.1 ± 0.2 Wm^−1^K^−1^ (about 19 times that of pure silicone rubber). Jin et al. [60] prepared a series of epoxy resin/aluminum nitride/Ni composites with different particle sizes of Ni powder. The results show that the thermal conductivity of composites is highest, which is 1.474 Wm^−1^K^−1^, higher than that of epoxy resin/aluminum nitride composites when 2 vol% Ni powders with particle size of 1 µm is added.

#### 3.1.3. Ceramics

Ceramics, including Al_2_O_3_ [47,61], AlN [62], SiC [63], BN [64,65], etc., are considered to be competitive candidates to improve the thermal conductivity of composite polymers.

Feng et al. [66] obtained polyolefin elastomer (POE)/BN composites using traditional two-roll milling method, in which the BN flakes is oriented horizontally. The laminated POE/BN bulks were further processed by hot-pressing method and followed by the mechanical cutting in the direction perpendicular to the BN flakes aligning. The resultant vertically oriented BN flakes composites have a high through-plane thermal conductivity (6.94 Wm^−1^K^−1^) and a BN loading of 43.75 vol%. Han et al. [67] constructed a nacre-mimetic 3D network of BN through bidirectional freezing and freeze-drying technique. The thermal conductivity of BN/epoxy resin composites prepared by infiltration method is up to 6.07 Wm^−1^K^−1^ when the content of BN is 15 vol%. Cheng et al. [68] modified the surface of SiC nanowires onto natural rubber by chemical grafting method, and then prepared composites by freeze-casting method, which will help to reduce phonon scattering. The thermal conductivity of the composites with 8.89 vol% filling can be as high as 0.856 Wm^−1^K^−1^.

### 3.2. Strategies to Enhance the Thermal Conductivity

Adding the thermally conductive fillers to polymer matrix will inevitably produce polymer-filler and filler-filler interfaces, so that a large amount of phonon scattering will be generated due to the mismatch of phonon vibration mode [3]. In this case, the thermal conductivity of thermally conductive polymer composites is far lower than the expected value. Therefore, eliminating or reducing the phonon scattering caused by the interfaces is the key to improve the thermal conductivity of thermally conductive polymer composites. By modifying the surface of fillers to increase the interaction between filler and polymer, the phonon scattering caused by the filler-matrix interfaces can be effectively reduced [69]. The popular approaches for surface modification of fillers include physical adsorption and chemical covalent bonding [70]. Shen et al. [71] successfully coated dopamine on the BN surface (PDA@BN) by chemical reaction. The dispersion uniformity of filler was improved and the interaction with polyvinyl alcohol (PVA) matrix was enhanced. As a result, the thermal conductivity of PDA@BN/PVA composites is higher than that of pristine BN composites; when the content of BN is 30 vol%, the thermal conductivity of PDA@BN/PVA composite is as high as 8.8 Wm^−1^K^−1^. Similarly, An et al. [5] constructed a 3D network of BN/reduced graphene oxide (BN/rGO) covalent bonding through surface modification and ice-templating self-assembly, which can significantly reduce the interfacial thermal resistances caused by polymer-filler and filler-filler interfaces. The BN/rGO/nature rubber (BN/rGO/NR) composite exhibits a high through-plane thermal conductivity of 1.28 Wm^−1^K^−1^ with the filler loading of only 4.9 vol%.

**Figure 6:**
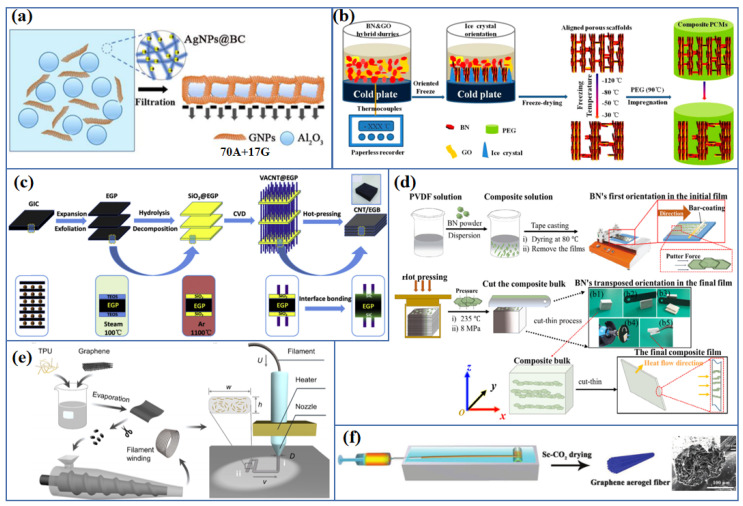
Illustration of approaches to construct an interconnected network of fillers by (**a**) vacuum-assisted layer-by-layer self-assembly strategy [47] (Copyright (2020) with permission from Elsevier B.V.), (**b**) ice-templating self-assembly strategy [84] (Copyright (2018) with permission from American Chemical Society), (**c**) CVD [85] (Copyright (2016) with permission from Elsevier Ltd.), (**d**) mold pressing [80] (Copyright (2019) with permission from Elsevier Ltd.), (**e**) 3D printing [86] (Copyright (2021) with permission from American Chemical Society) and (**f**) electrospinning [79] (Copyright (2018) with permission from Wiley Periodicals, Inc.).

It. is an effective way to reduce the phonon scattering caused by the filler-filler interface by designing the microstructure of composite and constructing an interconnected network of fillers as a heat conduction pathway [52]. In recent years, various approaches including vacuum-assisted layer-by-layer self-assembly [72], ice-templating self-assembly [73,74], chemical vapor deposition (CVD) [75], 3D printing [76,77,78], electrospinning [6,79], mold pressing [66,80], etc., have been reported to build interconnected filler networks (Figure 6). Vacuum-assisted layer-by-layer self-assembly is a process of solid–liquid separation driven by negative pressure. In this process, the sheet-like filler is stacked layer by layer and tightly connected, which is widely used in the preparation of interconnected filler network [55,81,82]. Feng et al. [7] reported that the flexible paper-like polymer-based thermal interface material prepared by simple vacuum-assisted filtration method have high in-plane thermal conductivity (39.27 Wm^−1^K^−1^ for NR/GNPs composite, and 11.82 Wm^−1^ K^−1^ for NR/BN composite). The thermal management capability of the resulting films is better than that of commercial thermally conductive NG papers and copper foils. Feng et al. [47] further prepared a flexible paper-like AgNPs@bacterial cellulose (BC)/Al_2_O_3_/GNPs composite film. This film has a high through-plane thermal conductivity of 9.09 Wm^−1^K^−1^ through a facile vacuum-assisted filtration method (Figure 5a), in which the heat transfer channel is provided by a single layer Al_2_O_3_ particles assisted by GNPs. Zeng et al. [83] fabricated an interconnected network composed of BN nanotubes (BNNTs) and cellulose nanofibers (CNFs) by a combination of ultrasonic dispersion and vacuum-assisted filtration. The obtained BNNTs/CNF composite has anisotropic thermal conductivity (21.39 Wm^−1^K^−1^ for in-plane direction and 4.71 Wm^−1^K^−1^ for through-plane direction).

Ice-templating self-assembly technology uses the highly anisotropic solidification behavior of the water in the orientation temperature field as a template to control the orientation of fillers, which has been widely used to construct 3D interconnected filler networks [87]. Xue et al. [88] prepared a GNPs aerogel by the means of ice-templating self-assembly and subsequent freeze-drying process, using melamine foam and CNFs as the co-media. After impregnating paraffin wax (PW), the composite displayed a high thermal conductivity of 1.42 Wm^−1^K^−1^ (increasing by 4.07 times compared with neat PW) at a low filler loading of 4.1 wt%. Yang et al. [84] constructed GO/BN hybrid porous scaffolds (HPSs) with 3D aligned network structure through unidirectional ice-templating self-assembly strategy, and then prepared polyethylene glycol (PEG)/HPSs composites by vacuum-assisted infiltration. When the BN loading is 28.7 wt%, the thermal conductivity of composites is 3.18 Wm^−1^K^−1^, which shows excellent solar-to-electric energy conversion performance. Yao et al. [74] constructed a 3D porous skeleton composed of stacked BN and reduced rGO (BN/rGO) by ball milling and subsequent the combination of ice-templating self-assembly and infiltrating methods. The obtained BN/rGO/epoxy composites possessed high though-plane thermal conductivity of 5.05 Wm^−1^K^−1^ at a filler loading of 13.16 vol%.

3D printing is an emerging technology, which can construct programmed microscopic and macroscopic structures [89,90], to realize the orderly stacking of thermally conductive fillers. Guo et al. [86] used 3D printing technology to prepare graphene/thermoplastic polyurethane (PU) composites. The orientation of graphene was realized by controlling printing parameters, and the resulting composites had a through-plane thermal conductivity of up to 12 Wm^−1^K^−1^. Chemical vapor deposition (CVD) and mold pressing assisted electrospinning are promising technologies for the fabrication of oriented interconnection filler network [6,91]. Qin et al. [85] constructed a 3D hierarchical CNT/exfoliated graphite block (CNT/EGB) by CVD on the surface of silicon oxide (SiO_2_)-coated exfoliated graphite plate, and then by hot-pressing procedure. The maximum through-plane thermal conductivity of CNT/EGB is up to 38 Wm^−1^K^−1^. Gu et al. [92] prepared BN/polyimide (PI) composites by in situ polymerization, electrospinning and subsequent hot pressing. The thermally conductivity, dielectric constant and dielectric loss tangent of BN/PI composites with 30 wt% BN is 0.696 W/m K, 3.77, and 0.007, respectively.

## 4. Thermally Conductive PCM (Thermal-Storage Thermal Management Material)

Thermal storage can be realized by sensible heat energy-storage technology, latent heat energy-storage technology and chemical thermal energy-storage technology [93]. Sensible heat energy-storage technology is based on the rise and fall of the temperature of storage material to store or release thermal energy, which strongly depends on the specific heat capacity of energy-storage material. The commonly used storage materials involve water, molten salt, pebbles, cement and so on. The sensible heat energy-storage technology is relatively simple, but in the process of storing and releasing thermal energy, the temperature fluctuates greatly, and the energy-storage density is usually low [94]. Chemical thermal energy-storage technology stores thermal energy through chemical endothermic reaction and releases thermal energy through exothermic reaction. It has the superior advantage of high energy-storage density, but the technology is complex, and the cost is high. Hence, the applications of the above two technologies have limitations. Latent heat energy-storage technology (phase-change energy-storage technology) stores and releases thermal energy through the phase-change process, during which the temperature is nearly constant [95]. The technology has the advantages of high energy-storage density, flexible design, and rich variety of material, so it has the most practical development prospect.

There are many kinds of phase-change material (PCM), which could be divided into solid–solid PCM, solid–liquid PCM, solid–gas PCM and liquid–gas PCM according to the phase-change process [96,97]. Among them, the volume of solid–gas PCM and liquid–gas PCM changes greatly in the process of energy-storage and release, which is not conducive to practical applications. Because solid–solid PCM stores and releases thermal energy through the transformation of crystal forms, it does not produce any gas or liquid. However, the energy-storage density is not enough to meet the application requirements. It is worth noting that solid–liquid PCM, including inorganic solid–liquid PCM and organic solid–liquid PCM, has attracted extensive attention of researchers due to their high energy-storage density and rich types [98,99]. The advantages and disadvantages of these two kinds of solid–liquid PCM are summarized in Table 2.

Compared with inorganic solid–liquid PCM, organic solid–liquid PCM, such as paraffin wax (PW), polyethylene glycol (PEG), fatty acid (FA), avoid the problems of phase separation and high supercooling, and show excellent comprehensive performance, so they, have wide application prospects in thermal management [102]. The thermophysical properties of commonly used organic solid–liquid PCM are summarized in Table 3. However, to realize the practical application of organic solid–liquid PCM, it is necessary to solve the leakage problem in the process of phase change [94,103]. Additionally, for faster thermal response and more efficient thermal management, the thermal conductivity need be increased [104].

### 4.1. Shape-Stabilized Composite PCM

**Figure 7:**
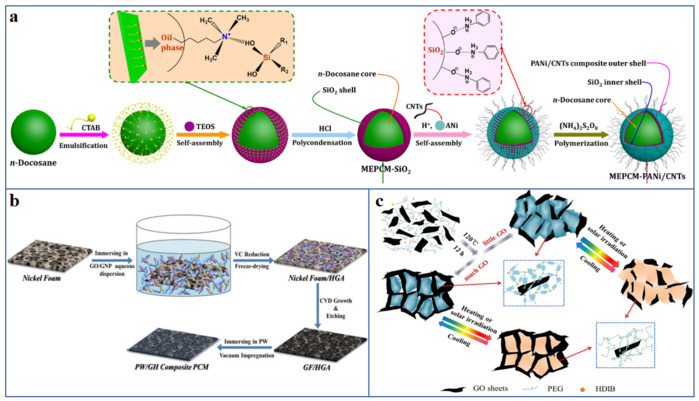
Illustration of preparation of shape-stabilized composite PCM by (**a**) encapsulated methods [110] (Copyright (2020) with permission from Elsevier B.V.), (**b**) introducing supporting material [111] (Copyright (2017) with permission from Elsevier B.V.) and (**c**) fabricating novel solid–solid composite PCM [112] (Copyright (2018) with permission from Elsevier B.V.).

So far, a variety of leak-proof strategies have been developed, such as the use of encapsulated methods, the introduction of supporting material, especially porous scaffolds, and the fabrication of novel solid–solid composite PCM [109]. Sun et al. [110] fabricated SiO_2_ inner shell by interfacial polycondensation onto the n-diocesan core, and then coated with polyaniline/CNT as an electrochemically active layer to construct microcapsules with layer-by-layer shell structure (Figure 7a). The PCM microcapsules have the advantages of good shape stability, phase-change enthalpy up to 140 J/g and excellent working reliability, which are expected to become promising candidates for thermal management material of supercapacitors. Yang et al. [111] constructed a 3D hybrid graphene aerogel (HGA) by self-assembly strategy and template-directed CVD method, and used it as supporting material to shape-stabilize the PW (Figure 7b). The resultant HGA/PW composite PCM exhibits enhanced thermal conductivity and improved shape stability, which has broad application prospects in the field of energy storage. Zhou et al. [112] synthesized a polyurethane-based solid–solid PCM by a facile solvothermal treatment, which was composed of PEG as working substance, hexamethylene diisocyanate biuret (HDIB) as crosslinking agent and rGO as light trapping agent (Figure 6c). Due to the existence of 3D crosslinking networks, the prepared rGO/PU composites still show outstanding shape stability when the temperature is higher than 100 °C.

#### 4.1.1. Encapsulated Composite PCM

Encapsulation by constructing core–shell structure is one of the common strategies to improve the shape stability of organic solid–liquid PCM, where the core is the PCM and the shell is a functional coating material. According to the obtained capsule diameter, the encapsulated method can be divided into macroencapsulated method (from 1 mm to 1 cm), microencapsulated method (from 1 μm to 1 mm) and nanoencapsulated method (<1 μm) [113]. Most of the capsules reported in the literature are in the micron range.

**Figure 8:**
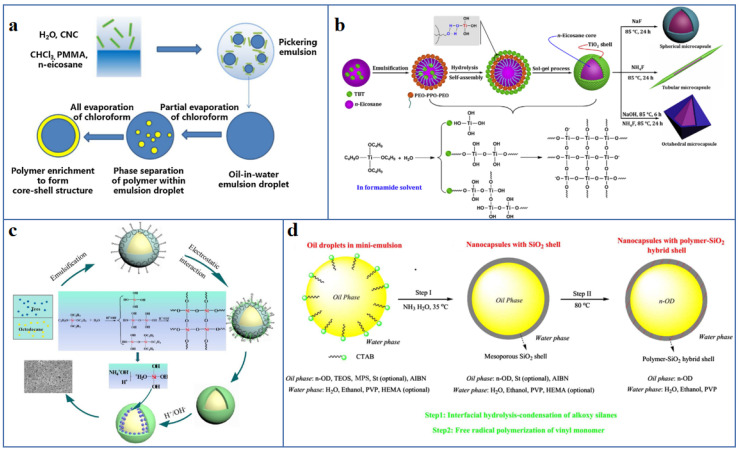
Various types of shell material have been applied to prepare PCM capsules: (**a**) polymers [125] (Copyright (2020) with permission from Elsevier Ltd.), (**b**) inorganic material [129] (Copyright (2019) with permission from Elsevier Ltd.), (**c**,**d**) polymer/inorganic hybrid material [123,130] (Copyright (2018) with permission from Elsevier Ltd. Copyright (2020) with permission from the American Chemical Society).

Shell material is the key to provide the stable encapsulation effect of the liquid PCM after solid–liquid phase transition, to ensure the cycle durability and no liquid leakage of PCM. Common functional coating material include polymers and inorganic material [114], such as polymethylmethacrylate (PMMA) [115], polystyrene (PS) [116], melamine formaldehyde (MF) [117], SiO_2_ [118,119], titanium dioxide (TiO_2_) [120], calcium carbonate (CaCO_3_) [121], etc. Polymer has been widely used in functional coating material because of its advantages of good chemical stability, excellent processability, good mechanical properties, satisfactory compatibility with organic solid–liquid PCM. The polymer was coated on the PCM cores using emulsion polymerization [122], interfacial polymerization [123] and in situ polymerization [124]. As shown in Figure 8a, Wang et al. [125] used Pickering emulsion-templating assisted solvent evaporation approach to prepare microencapsulated PCM with n-eicosane as the core and PMMA as the shell. The microcapsule shows a regular spherical shape with the size in the range of 5–10 μm, and it has good shape stability and great thermal reliability for high retention rate of melting enthalpy. Naikwadi et al. [126] coated the MF on n-tetradodecane core by two-step in situ polymerization to obtain the microencapsulated PCM. The results of repeatable heating–cooling cycles show that the shape does not change, and the thermal performance could be observed, displaying good shape stability and cycle durability. Unfortunately, the low thermal conductivity and the intolerant to high temperature of polymer/PCM microcapsules restrict their applications in some fields [113].

Inorganic shell material with high thermal conductivity and thermal stability are also widely used to prepare PCM microcapsules with stable shape [127]. Fang et al. [128], using tertbutyl orthosilicate (TEOS) as the precursor, synthesized PW/SiO_2_ microencapsulated composite PCM by sol–gel method. The encapsulation ratio of the microcapsules is 87.5%, the thermal stability and flame retardancy was improved. Even if the temperature is higher than the melting point of PW, and the leakage of PW also be suppressed. Liu et al. [129] synthesized the n-eicosane/TiO_2_ microcapsule suspension, and then introduced different crystallization promoters to induce the tubular, octahedral and spherical morphology of the microcapsules and the crystal structure of the TiO_2_ shell. The effective encapsulation ratio of spherical microcapsules is 75.7%, and the thermal conductivity of tubular microcapsules was the highest, which is 1.216 Wm^−1^K^−1^ (Figure 8b).

The inorganic shell is fairly brittle and almost incompatible with organic solid–liquid PCM, therefore, by introducing hybrid shell, microcapsules with excellent comprehensive performance such as enhanced thermal conductivity, good chemical stability and good mechanical toughness were prepared [131]. For example, Zhai et al. [130] constructed n-octadecane-based microencapsulated PCM with hybrid shell by in situ polymerization of MF and the hydrolysis-polycondensation of TEOS on n-octadecane (Figure 8c). The double-encapsulated microcapsules show excellent shape stability, enhanced thermal conductivity and cycle durability. Zhu et al. [123] prepared a hybrid shell by interfacial hydrolysis-polycondensation of TEOS on n-octadecane core, followed by the radical polymerization of styrene and hydroxyethyl methacrylate (HEMA) (Figure 8d). The nanoencapsulated PCM with PS-SiO_2_ hybrid shell and PHEMA-SiO_2_ hybrid shell display improved shape stability, enhanced thermal conductivity, and good mechanical properties.

#### 4.1.2. Supporting Material

**Figure 9:**
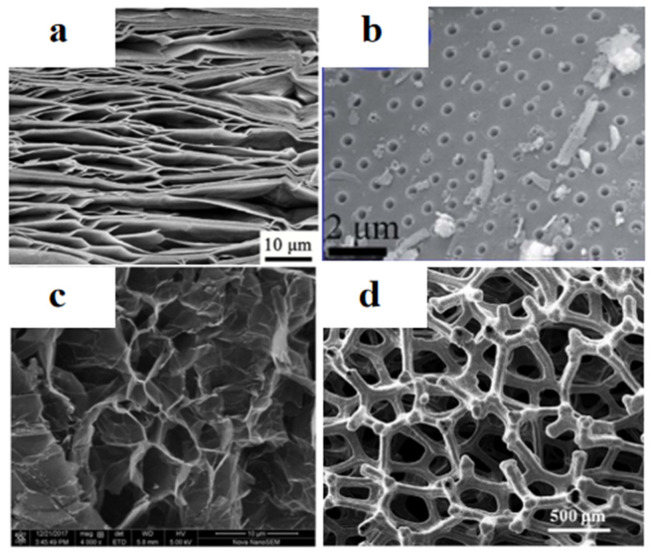
SEM images of (**a**) EVM [147] (Copyright (2021) with permission from Elsevier B.V.), (**b**) diatomite [133] (Copyright (2015) with permission from Royal Society of Chemistry), (**c**) EG [43] (Copyright (2018) with permission from Elsevier Ltd.), (**d**) 3D porous diamond foam [137] (Copyright (2018) with permission from Elsevier Ltd.).

It is an effective way to fabricate shape-stabilized composite PCM by introducing supporting material into organic solid–liquid PCM through physical mixing. Material with the following two characteristics are considered to be promising supporting material: (1) Material with porous structure, such as expanded vermiculite (EVM) [132], diatomite [133], EG [134], active carbon (AC) [135], 3D porous frameworks [136,137], etc., can absorb and contain PCM through the capillary force and surface tension of these pores. The microstructure of some of this material is shown in Figure 9. (2) Material that can form strong interactions with PCM, which limit the movement ability of the PCM molecular chains, such as hydrogen bond force [138].

For the first kind of supporting material, Jin et al. [139] prepared stearic acid (SA)/palmitic acid (PA)/diatomite ternary composite PCM by vacuum impregnation, and the obtained PCM exhibited excellent thermal and chemical stability. When SA/PA/diatomite composites are used in asphalt pavements, it has promising cooling ability, specifically, the maximum surface cooling could reach 8.11 °C. Song et al. [140] synthesized dodecane/EG composite PCM by vacuum infiltration method. The results manifested that when the EG content was higher than 16 wt%, the leakage of composite PCM could not be observed, which indicated that the shape of composite PCM was stable due to the adsorption of EG pores. Wang et al. [1] pyrolyzed melamine diborate at 1400 °C in the N_2_ atmosphere, a flexible BN aerogel film with high porosity was designed. Then PW was introduced into the BN aerogel by vacuum impregnation. The resultant composite PCM possessed good shape stability and excellent temperature control ability, which is expected to serve as a smart thermal regulator for practical application. Ding et al. [141] prepared PEG/rGO composite PCM by vacuum infiltration. The results indicated that the loading content of PEG is as high as 96.6%, much higher than that prepared by hydrothermal reduction method, and because of the encapsulation effect of rGO aerogel, there is no leakage of liquid in PEG/rGO composites, displaying good shape stability.

For the second kind of supporting material, Shi et al. [142] prepared composite PCM with poly (ethylene oxide) (PEO) as PCM and surface-carboxylated cellulose nanofibers (CNFs) as supporting material by casting and drying. The prepared composite films have good solid–solid phase-change characteristics and improved mechanical properties. As one of the most widely used PCM, PEG has hydroxyl groups in its molecular chain, which can form strong hydrogen bonds with cellulose [143], GO [144], etc., and can be served as a supporting material to overcome the problem of PEG leakage. For example, Qi et al. [145] introduced GO sheets PEG to fabricate composite PCM with stable shape. Due to the restriction of high specific surface area GO sheets on the movement of PEG molecular chains, GO/PEG with the maximum PEG content of 96 wt% has no leakage at the temperature up to 150 °C. Zhou et al. [146] developed a facile method based on thiolene click chemistry and solvent exchange to fabricate solid–solid PCM. Accordingly, cellulose nanocrystal (CNC) hydrogel with porous structure was used as supporting material to stabilize the shape of PEG. Due to the synergistic effect of pores adsorption and hydrogen bonding between PEG and CNC, the composite PCM possessed excellent shape stability.

#### 4.1.3. Solid–Solid Composite PCM

It is noted that adding supporting material or constructing core–shell structure is not suitable for some applications, such as food refrigeration and medicine packaging. Therefore, the preparation of solid–solid PCM by chemical synthesis to overcome the leakage problem has attracted extensive attention of researchers. Recently, polyurethane (PU) as the representative of new solid–solid PCM is mainly synthesized by block polymerization of small phase-change molecules and supporting material [148]. Harlé et al. [149] synthesized cross-linked PU by solvent-free one-step bulk polymerization with PEG of different molecular weight. The resultant cross-linked PU still has strong crystallization ability and displays the characteristics of solid–solid phase change. Kong et al. [150] prepared cross-linked PU/lauric acid composite PCM through a brief thermal curing reaction, in which the cross-linked PU was used not only as a supporting material, but also as PCM. The results reveal that the phase-change enthalpy of PCM is as high as 131 J g^−1^, which shows good thermal reliability and shape stability. In addition, new solid–solid PCM were synthesized by chemical grafting method, and PCM were grafted onto high-melting-point material. Cao et al. [151] fabricated a novel solid–solid PCM based on hexadecyl acrylate (HDA) and GO sheets by radical polymerization, in which HAD was grafted onto GO sheets by covalent bond. The composite PCM has amazing shape stability and no leakage when the temperature is 80 °C.

### 4.2. Strategies to Enhance the Thermal Conductivity of PCM

**Figure 10:**
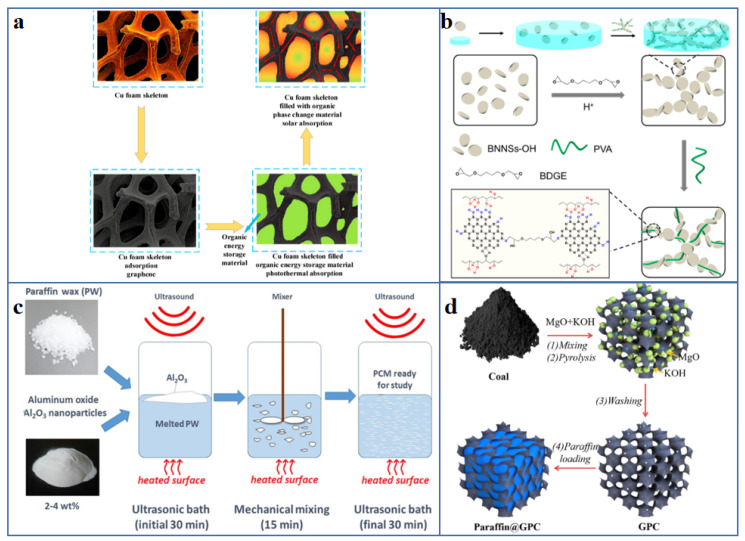
Schematic diagram of preparation of thermally enhanced composite PCM by introducing different types of thermally conductive fillers: (**a**) metals [152] (Copyright (2019) with permission from Elsevier Ltd.), (**b**,**c**) ceramics [153,154] (Copyright (2019) with permission from American Chemical Society. Copyright (2019) with permission from Elsevier Ltd.), (**d**) 3D porous frameworks [155] (Copyright (2019) with permission from Elsevier B.V.).

The main thermally conductive fillers used to enhance the thermal conductivity of the composite PCM are the same as those used in the fabrication of above-mentioned thermally conductive polymer composites, so the fillers are not discussed repeatedly in this section (Figure 10). However, several examples will be given to illustrate the application of various thermally conductive fillers in enhancing the thermal conductivity of composite PCM.

Kim et al. [156] prepared composite PCM by blending Ag nanoparticles (AgNPs) with molten PW under ultra-sonication. The results show that the introduction of AgNPs can improve the thermal conductivity of Ag/PW composites. Specifically, when the AgNPs content is in the range of 0.5–2.0 wt%, the thermal conductivity of Ag/PW with the AgNPs size of 9 nm, 65 nm, and 300 nm, is 0.270–0.321 Wm^−1^K^−1^, 0.273–0.305 Wm^−1^K^−1^ and 0.280–0.291 Wm^−1^K^−1^, respectively. Qian et al. [133] fabricated composite PCM with enhanced thermal conductivity by compounding PEG and AgNPs-modified diatomite. When the AgNPs content is only 7.2 wt%, the thermal conductivity of ternary composite PCM is as high as 0.82 Wm^−1^K^−1^, which is 127% higher than that of PEG/diatomite. Cheng et al. [157] reported a PW@vanadium dioxide (VO_2_) composite PCM, in which PW as the core (solid–liquid PCM), VO_2_ (solid–solid PCM) as the shell. The resultant composite PCM exhibited excellent shape stability and thermal conductivity of 1.53 Wm^−1^K^−1^. Jiang et al. [153] prepared an elastic dual-crosslinked BN network (BNN), in which covalent bonds act as permanent crosslinking points and hydrogen bond act as temporary crosslinking points. The PEG/BN/BNN ternary composites were fabricated by vacuum-assisted infiltration, and its thermal conductivity is 0.67 Wm^−1^K^−1^. Akhmetov et al. [154] prepared composite PCM by dispersing nano-Al_2_O_3_ particles into high-melting-point PW (H-PW) and low melting point PW (L-PW). The measurement results of laser flash apparatus reveal that the thermal diffusivity of H-PW/Al_2_O_3_ and L-PW/Al_2_O_3_ increased by 25% and 40%, respectively, when the loading content of Al_2_O_3_ reaches 4 wt%. Li et al. [158] exfoliated natural flake graphite to obtain ultra-thin graphite sheets (UGSs) by ultrasonication-milling and subsequent supercritical CO_2_ assisted exfoliation. The composites of stearic acid (SA) and UGSs was prepared by vacuum impregnation. The thermal conductivity of SA/UGSs is 2.691 Wm^−1^K^−1^, which is 10 times higher than that of pure SA. Harish et al. [159] fabricated lauric acid (LA)-based composite PCM by liquid phase exfoliation of multilayer GNPs. The obtained LA/GNPs composite showed high thermal conductivity of 0.489 ± 0.01 Wm^−1^K^−1^ at a relatively low GNPs loading (1.0 vol%).

The 3D porous framework not only improve the leakage problem of organic solid–liquid PCM through the physical adsorption of capillary force and surface tension, but also improve the thermal conductivity of the composite PCM significantly and therefore accelerate the thermal response rate because its 3D interconnected networks could provide heat conduction channels. Min et al. [160] constructed an anisotropic graphene aerogels through directional-freezing polyamic acid salt/GO slurries, followed by graphitization, and then impregnated with PW was to fabricate composite PCM. The results show that the composite PCM have good shape stability and high anisotropic thermal conductivity, which are 2.68 Wm^−1^K^−1^ along the through-plane direction and 8.87 Wm^−1^K^−1^ along the in-plane direction. Zhu et al. [155] reported that the hierarchical graphitic porous carbon (GPC) derived from coal can be obtained by carbonizing the mixture of coal, MgO and KOH powder at 900 °C in argon atmosphere for 1 h. Then PW was impregnated into GPC to fabricate shape-stabilized PW/GPC composites. When the capacity of PW is 90 wt%, the thermal conductivity of PW/GPC composites.is increased by 0.58 Wm^−1^K^−1^, and it maintains good shape stability at 65 °C.

## 5. Thermal Management Applications

Due to the pursuit of high-quality life, the demand for advanced, intelligent, and multi-functional electronic products continues to rise. Therefore, it is of great significance to solve the overheating and thermal safety problems of electronic devices. Therefore, it is essential to construct an effective heat conduction pathway or provide a cooling system with high heat storage capacity to eliminate and dissipate heat in electronic devices, so as to maintain the temperature of electronic devices within the working temperature range, therefore to achieve the purpose of efficient and safe operation of electronic devices [161]. As mentioned above, polymer-based thermal management material has excellent comprehensive properties that can meet the requirements of effective heat dissipation. Its applications in electronic devices are mainly served as heat-spreader [162,163] and thermal interface material (TIM) [7,164,165].

For the heat-spreader, it needs high in-plane thermal conductivity to conduct the heat from the point heat source to a large surface area, and then dissipate the heat through heat convection and radiation. Recently, some carbon-based thermally conductive film material with ultrahigh in-plane thermal conductivity and low thermal expansion coefficient have been reported. For example, the graphene film annealed at 3000 °C and compressed at 300 MPa has an extremely high in-plane thermal conductivity of 1940 ± 113 Wm^−1^K^−1^ [166]. Shen et al. [167] fabricated a graphene film by direct evaporation of GO suspension at 50–60 °C and then thermal annealing at 2000 °C for the reducing and graphitizing of GO film. The graphene film exhibits high in-plane thermal conductivity of ~1100 Wm^−1^K^−1^. However, the mechanical properties, especially elongation at break and flexibility of carbon-based thermally conductive film material, are generally unsatisfactory, which makes it difficult to contact well with electronic devices. Consequently the thermally conductive polymer composites with high in-plane thermal conductivity discussed above are more suitable for heat-spreader material, such as BNNS/poly(diallyl dimethyl ammonium chloride) composites [82] with ~200 Wm^−1^K^−1^ in-plane thermal conductivity, BNNTs/CNFs composites with 21.39 Wm^−1^K^−1^ in-plane thermal conductivity [83], NR/GNPs composites [7] with 39.27 Wm^−1^K^−1^ in-plane thermal conductivity, and rGO/poly (vinylidene fluoride-co-hexafluoropropylene) composites [168] with 19.5 Wm^−1^K^−1^in-plane thermal conductivity, etc.

For TIM, the total thermal resistance (*R*) of TIM can be expressed as [165]:

$$R=\frac{BLT}{K_{TIMs}}+R_{c1}+R_{c2}$$

where *BLT* and *K_TIMs_* are the thickness and the thermal conductivity of TIM, *R_c_*_1_ and *R_c_*_2_ are the contact thermal resistances at interfaces. Therefore, increasing the thermal conductivity, especially through-plane thermal conductivity, is to accelerate longitudinal heat transfer and reduce the air layer at interfaces (the thermal conductivity of air layer is as low as 0.02 Wm^−1^K^−1^) is the key to decrease the *R* value for achieving efficient heat dissipation. Flexible polymer-based composites could fit well with devices, so the above-discussed high through-plane thermally conductive polymer composite is a promising candidate for serving as TIM to realize the thermal management of electronic devices, such as POE/BN composites [66] with the through-plane thermal conductivity of 6.94 Wm^−1^K^−1^, AgNPs@BC/Al_2_O_3_/GNPs composites [47] with the through-plane thermal conductivity of 9.09 Wm^−1^K^−1^, graphene/PU composites with the through-plane thermal conductivity of 12 Wm^−1^K^−1^ [86], BN/poly (vinylidene fluoride) composites [80] with the through-plane thermal conductivity of 3.5 Wm^−1^K^−1^, EG/epoxy composites with the through-plane thermal conductivity of 4.14 Wm^−1^K^−1^ [49], etc.

Currently, commercial TIM mainly consists of thermal greases, thermal gels, PCM, thermal pads, thermal adhesives, etc. These kinds of TIM have some disadvantages, such as thermal greases are prone to phase separation and phase migration, thermal gels and thermal adhesives require additional curing process, BLT of thermal pads is high, the thickness is uneven, and PCM has leakage problem. PCM have great energy-storage capability, cycle durability and special phase-change characteristics. In the process of solid–liquid conversion, they can not only effectively fill the air layer, but also store a large amount of heat. Through the methods summarized in Chapter 4, the leakage problem of PCM is solved, and the thermal conductivity is improved, which can be used as TIM and heat-spreader [169,170].

## 6. Conclusions and Outlook

This review systematically introduces two typical polymer-based thermal management materials: thermal-conduction thermal management material and thermal-storage thermal management material. The advanced structural design and processing strategies of polymer-based thermal management material with excellent performance reported in recent years are summarized.

For thermal-conduction thermal management material, improving thermal conductivity is the key to ensure their efficient heat dissipation. On the one hand, the intrinsic thermally conductive polymers with high thermal conductivity can be obtained by changing the structure of polymer molecular chain or increasing the order of molecular chain arrangement by external force field. On the other hand, the thermally conductive polymer composites with high thermal conductivity can be prepared by constructing an interconnected network of thermal conductive fillers. For thermal-storage thermal management material based on solid–liquid PCM, it is significant to improve their shape stability and thermal conductivity for efficient heat dissipation. Using various effective encapsulation strategies (building core–shell structure, introducing supporting material, synthesizing solid–solid PCM) and constructing heat conduction pathway, the composite PCM with efficient heat dissipation was obtained.

However, there are still some issues worthy of further consideration in the development and large-scale production of high-performance polymer-based thermal management material. (1) Systematic research should be carried out to clarify the relationship between the microstructure, filler network, thermal conductivity of composite material, and the actual heat dissipation effect, to reasonably design the structure and realize different degrees of dissipation purposes. (2) For polymer-based thermal management material, not only the thermal conductivity should be increased, but also the electrical insulation, corrosion resistance, flame resistance, aging resistance, and long-term stability should be improved. (3) New thermal conductive fillers or filler modification techniques should be developed to obtain perfectly interconnected thermal pathways by simple composite methods. (4) It is necessary to further research and develop a facile and scalable processing strategy for high-performance polymer-based thermal management material with low filling content.

## Figures and Tables

**Table 1:** Thermal conductivities of commonly used thermally conductive fillers [13,15,37].

Fillers		Thermal Conductivity (Wm^−1^K^−1^)
Metals	Silver (Ag)	~420
	Cupper (Cu)	401
	Aluminum (Al)	237
	Nickel (Ni)	158
	Zinc (Zn)	121
Carbon material	Carbon fibers (CFs)	300–1000
	Carbon nanotubes (CNTs)	2000–6000
	Graphite	100–400
	Graphene	5300
Ceramics	BN	250–300
	Aluminum nitride (AlN)	300
	Silicon carbide (SiC)	120
	Aluminum oxide (Al_2_O_3_)	30–40

**Table 2:** Advantages and disadvantages of organic solid–liquid PCM and inorganic solid–liquid PCM [94,100,101].

Solid–Liquid PCM	Advantages	Disadvantages
Inorganic solid–liquid PCM	High energy-storage densities High thermal conductivities Low costs	4. Large supercooling 5. Phase separation
Organic solid–liquid PCM	High energy-storage densities Wide range of phase change temperature for convenient use Isothermal characteristics No phase separation Low or negligible supercooling Non-toxicity and non-corrosion Desirable thermal and chemical stability for long-term use Abundant natural resources	9. Poor shape stability (leakage during phase transition) 10. Low thermal conductivity

**Table 3:** The thermophysical properties of commonly used organic solid–liquid PCM [105,106,107,108].

PW	Molecular Formula	T_m_ (°C)	T_c_ (°C)	∆H (J/g)
n-Hexadecane	CH_3_(CH_2_)_14_CH_3_	18–19	17	237
n-Octadecane	CH_3_(CH_2_)_16_CH_3_	28	25	242
n-Eicosane	CH_3_(CH_2_)_18_CH_3_	36–37	31	247
n-Docosane	CH_3_(CH_2_)_20_CH_3_	42–45	43	157
n-Tetracosane	CH_3_(CH_2_)_22_CH_3_	50–51	48–49	160
n-Hexacosane	CH_3_(CH_2_)_24_CH_3_	56	53–54	255
PEG	Molecular weight (g/mol)	Tm (°C)	Tc (°C)	∆H (J/g)
PEG400	400	3.2	−24	91.4
PEG1000	1000	32.0	28	149.5
PEG2000	2000	51.0	35	181.4
PEG4000	4000	59.7	22	189.7
PEG10000	10,000	66.0	38	189.6
PEG20000	20,000	68.7	38	187.8
FA	Molecular formula	Tm (°C)	Tc (°C)	∆H (J/g)
Caprylic acid	CH_3_(CH_2_)_6_COOH	16–17	-	148–149
Capric acid	CH_3_(CH_2_)_8_COOH	30–32	-	152.7–155.46
Lauric acid	CH_3_(CH_2_)_10_COOH	42–44	39–42	175–190
Myristic acid	CH_3_(CH_2_)_12_COOH	51.5–58	51–52	178.14–210.7
Palmitic acid	CH_3_(CH_2_)_14_COOH	61–64	58–60.38	185.4–212.1
Stearic acid	CH_3_(CH_2_)_16_COOH	65–70	66–67	198.8–258.98
